# Pyrolysis for Nylon 6 Monomer Recovery from Teabag Waste

**DOI:** 10.3390/polym12112695

**Published:** 2020-11-16

**Authors:** Soosan Kim, Nahyeon Lee, Jechan Lee

**Affiliations:** 1 Department of Environmental Engineering, Ajou University, 206 Worldcuo-ro, Suwon 16499, Korea; ksoosan@ajou.ac.kr; 2 Department of Environmental and Safety Engineering, Ajou University, 206 Worldcuo-ro, Suwon 16499, Korea; skgus@ajou.ac.kr; 3 Department of Energy Systems Research, Ajou University, 206 Worldcuo-ro, Suwon 16499, Korea

**Keywords:** polymer recycling, thermochemical process, waste valorization, nylon, biofuels

## Abstract

In this work, we used pyrolysis to treat teabag waste (TBW). Changes in the pyrolysis temperature affected the composition and yield of the products. For example, more non-condensable gases and less char were produced with an increase in the pyrolysis temperature. Pyrolysis conducted under a nitrogen environment yielded caprolactam at temperatures between 400 and 700 °C. An increase in the pyrolysis temperature from 400 to 500 °C increased the caprolactam yield from 3.1 to 6.2 wt.%. At 700 °C, the yield decreased to 4.6 wt.%. The highest caprolactam yield (i.e., 6.2 wt.% at 500 °C) was equivalent to 59.2 wt.% on the basis of the weight of the non-biomass part of the TBW. The pyrolytic products other than caprolactam (e.g., combustible gases, pyrolytic liquid, and char) can function as fuels to supply energy during pyrolysis in order to increase and maintain the temperature. The higher heating values (HHVs) of the combustible gases and pyrolytic liquid produced at 500 °C were 7.7 and 8.3 MJ kg^−1^, respectively. The HHV of the char produced at 500 °C was 23 MJ kg^−1^, which is comparable to the HHV of coal. This work will help to develop effective pyrolysis processes to valorize everyday waste by recovering value-added chemicals such as polymer monomers and by producing alternative fuels.

## 1. Introduction

Nylon 6 is a commonly-used polymer in the fiber and textile industries due to its high resistance to heat and wear, good mechanical properties, and processability [1]. Its applications include electrical equipment, car parts [2], food packaging materials [3,4], flooring, reinforced rubber, and apparel [5]. For example, nylon 6 is commonly used to make teabags [6]. Nylon 6 is synthesized via the ring-opening polymerization of caprolactam [7,8,9]; hence, nylon 6 is also known as polycaprolactam. The global market size of caprolactam in 2016 was $11.55 billion USD with an estimated compound annual growth rate of 5.2% from 2012 to 2022 [10].

Caprolactam is made from petroleum-derived chemicals, either cyclohexane or phenol, via complicated multi-stage processes [11]. Caprolactam recovery via the depolymerization of used nylon 6 has been investigated as a method to reduce the environmental burden of caprolactam production [12,13,14]. These methods are high-pressure processes conducted in the liquid phase in the presence of homogenous catalysts. However, they are only applicable for pure nylon 6 and are difficult to apply to mixed materials such as waste.

Tea is one of the most consumed beverages in the world [15]. According to the Food and Agriculture Organization (FAO) of the United Nations, approximately 4.8 million tons of tea were consumed in 2013, and tea consumption is expected to grow at a rate of 3% until 2023 [16]. Heavy tea consumption leads to the generation of a large amount of tea waste. Teabag waste (TBW) is representative of tea waste because teabags are widely used due to their convenience. Teabags contain harmful chemicals such as flavonoids, phenolic compounds, alkaloids, and methyxanthines [15] and do not easily degrade [17]. In addition, teabags are commonly made of plastic (e.g., nylon). The plastic contained in TBW is not biodegradable and thus remains in the environment for a long time [18], causing various environmental problems such as bioaccumulation [19], shortened plant life [20], and microplastic release [21]. Landfills are commonly used to dispose of waste teabags [22]. Landfilling TBW can contaminate the surrounding soil and groundwater and emit landfill gas, which is an explosion hazard and which contributes to global warming [23]. Incineration can be used to dispose of TBW; however, it emits air pollutants such as particulate matter, dioxins, and polycyclic aromatic hydrocarbons (PAHs) [24,25] that are harmful to the environment and human health [26].

Pyrolysis can produce pyrolytic gas, pyrolytic oil, and char from different waste materials under an oxygen-free inert environment [27]. Pyrolytic gas is a gaseous mixture primarily comprising hydrogen (H_2_), carbon monoxide (CO), carbon dioxide (CO_2_), and C1 to C3 hydrocarbons [28]. Pyrolytic oil is a complex mixture of condensable species [29]. It is also called bio-oil when biomass is the feedstock and is considered an alternative liquid fuel [30]. Char is a carbonized residue that can be used as an alternative solid fuel (i.e., charcoal) [31].

Although pyrolysis is often exploited to produce these alternative fuels, it has rarely been used to recover polymer monomers from waste materials. In this regard, we sought a pyrolysis process to recover value-added polymer monomers (e.g., caprolactam) from TBW. Gaseous, liquid, and solid pyrolytic products obtained from TBW were identified and analyzed. The results of this study will help to develop sustainable processes to recover polymer monomers from waste materials.

## 2. Materials and Methods

### 2.1. Feedstock

Teabags were purchased from a local supermarket located in Suwon-si, Korea. The teabags were brewed in water at 90 °C for 10 min and then thoroughly washed. The washed teabags were then dried at 60 °C for 48 h. The dried teabags (Figure S1) were considered TBW and used as the feedstock in this study. The weight ratio of biomass part to non-biomass part (Figure S1) was 5:1.

### 2.2. Feedstock Characterization

Proximate and ultimate analyses of the TBW were conducted according to methods reported elsewhere [32]. Composition of the biomass part of the TBW was analyzed using the method reported by Ho and co-workers [33]. Thermogravimetric analysis (TGA) of the TBW was conducted under nitrogen (N_2_) atmosphere (N_2_ flow rate: 60 mL min^−1^) from 30 to 1000 °C (heating rate: 10 °C min^−1^) using a thermal analyzer (model: STA449 F3; NETZSCH, Selb, Germany).

### 2.3. Pyrolysis Experiment

The reactor setup used to pyrolyze the TBW is depicted in Figure S2. The TBW was pyrolyzed as it was without separating the biomass part and non-biomass part and without milling (Figure S2). The pyrolyzer consisted of a tube furnace equipped with a temperature controller (HANTECH, Gunpo-si, Korea), a mass flow controller (MFC) (KOFLOC, Kyoto, Japan), and a cold trap (to collect condensable species) comprising an ice bath (−1 °C) and a dry ice/acetone bath (−55 °C). A quartz tube was located in the heating zone of the tube furnace. TBW was placed in the center of the quartz tube. The pyrolysis temperature was controlled by the temperature controller (heating rate: 230 °C min^−1^). The N_2_ gas flow rate (ultra-high purity; DK gas, Hwaseong-si, Gyeonggi-do, Korea) was controlled by the MFC to maintain a flow rate of 100 mL min^−1^.

### 2.4. Product Analysis

The non-condensable gases were identified and quantified in situ by a micro gas chromatograph (GC) (INFICON, Bad Ragaz, Switzerland) directly connected to the quartz tube outlet. The condensable species collected by the cold trap were analyzed by GC–mass spectrometry (GC–MS) (Agilent, Santa Clara, CA, USA). The specifications and analytical conditions of the micro GC and GC–MS are shown in Tables S1 and S2, respectively.

The higher heating value (HHV) of combustible gases was calculated based on the enthalpies of combustion of each gas [34]. The HHV of the pyrolytic liquid was measured using a bomb calorimeter. An empirical equation [35] was used to estimate the HHV of char, as follows:

HHV = 34.91*X*_C_ + 117.83*X*_H_ + 10.05*X*_S_ − 10.34*X*_O_ − 1.51*X*_N_ − 2.11*X*_Ash_

where *X*_C_, *X*_H_, *X*_S_, *X*_O_, *X*_N_, and *X*_Ash_ are the fraction of carbon, hydrogen, sulfur, oxygen, nitrogen, and ash of char (by weight).

## 3. Results and Discussion

Figure S1 shows that the TBW is composed of non-biomass and biomass parts. The non-biomass contained 0.6 wt.% of fixed matter, and the biomass part contained 18.7 wt.% of fixed matter. The non-biomass part consisted mostly of volatile matter (96.5 wt.%). The non-biomass part had less oxygen and more nitrogen than the biomass part. The difference in thermochemical and elemental compositions between the non-biomass and biomass parts is because the non-biomass part is composed of synthetic polymer materials [36]. Sulfur was not detected in the TBW.

**Figure 1:**
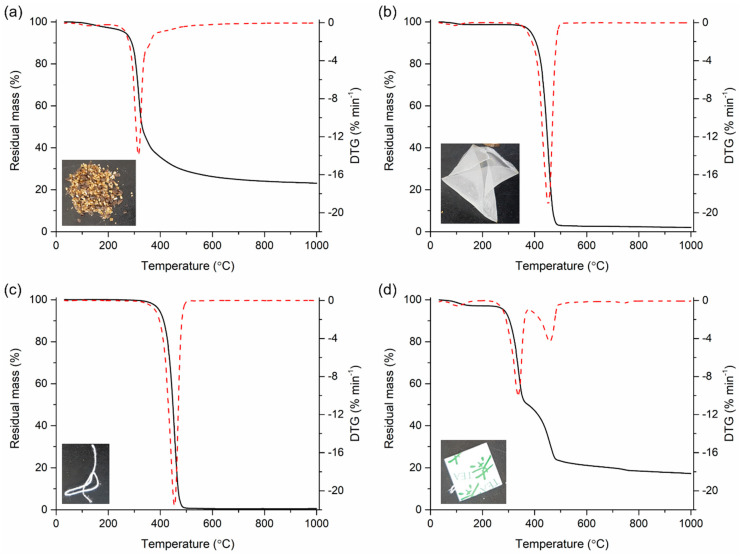
TG and DTG curves of components of the TBW: (**a**) biomass; (**b**) plastic bag; (**c**) thread; and (**d**) paper.

The TGA results of each component comprising the TBW are summarized in Figure 1. As seen in Figure S1, the TBW was composed of four different parts: biomass (i.e., brewed tea leaves), plastic bag containing the tea leaves, thread, and paper (the thread and paper for removal of teabag after brewing). Figure 1a shows the TG and DTG curves of the biomass. Major thermal degradation of the biomass was found between 200 to 500 °C. Approximately 20 wt.% of the biomass remained after the TGA, attributed to the content of fixed matter and ash (Table 1). The plastic bag and thread showed a similar trend in thermal degradation (Figure 1b,c). More than 98 wt.% of the plastic bag and thread was thermally degraded until 900 °C. The thermal degradation of the plastic bag and thread mostly occurred from 350 to 490 °C. Figure 1d presents two distinctive zones of thermal degradation of the paper. The first zone between 260 and 360 °C was associated with degradation of hemicellulose contained in the paper, and the second zone between 360 and 480 °C was associated with degradation of cellulose contained in the paper [37]. Other minor thermal degradation patterns shown in Figure 1d were likely ascribed to degradation of ink printed on the paper.

To ascertain how much of the TBW was transformed into an individual compound by pyrolysis, we wrote a mass balance between the feedstock (the TBW) and all the compounds identified in the pyrolytic products. The non-condensable gases were H_2_, CO, CO_2_, alkanes (e.g., methane (CH_4_), ethane (C_2_H_6_), and propane (C_3_H_8_)), and alkenes (e.g., ethylene (C_2_H_4_) and propylene (C_3_H_6_)). The condensable species were classified as phenolic compounds, benzene derivatives, PAHs and their derivatives, oxygenated compounds, and hydrocarbons.

**Figure 2:**
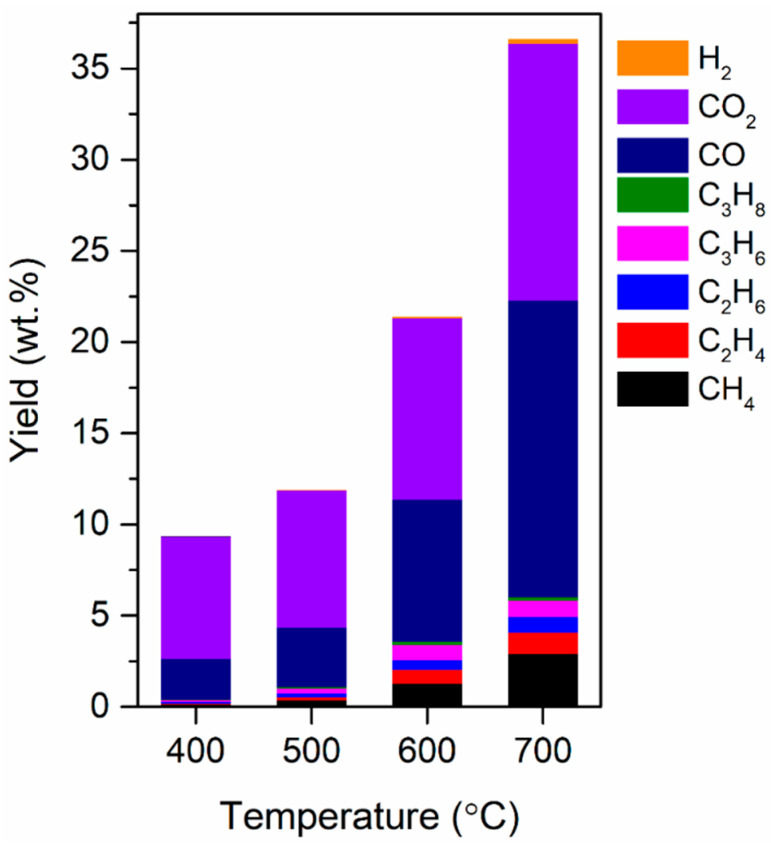
Yield of non-condensable gases (on the basis of the weight of the feedstock) produced via the pyrolysis of TBW at different pyrolysis temperatures. Mean values of replicates (*n* = 3) are reported with standard deviations of approximately 7%.

Figure 2 shows the yields of the non-condensable gases identified in the pyrolytic products (i.e., pyrolytic gases) obtained from the TBW as a function of the pyrolysis temperature. On a weight basis, CO and CO_2_ were the most prominent permanent gases produced by the pyrolysis of the TBW. The yields of both CO and CO_2_ increased as the pyrolysis temperature increased. The yields of alkanes and alkenes were not strongly dependent on the temperature. The H_2_ yield increased with an increase in the temperature. The total yield of non-condensable gases increased from 9.4 to 36.6 wt.% as the pyrolysis temperature increased from 400 to 700 °C. This could be because the cracking of condensable vapor via the homogeneous reactions taking place in the gas phase and the heterogeneous reactions taking place in the gas–solid phase are thermally enhanced at higher temperatures [38].

**Figure 3:**
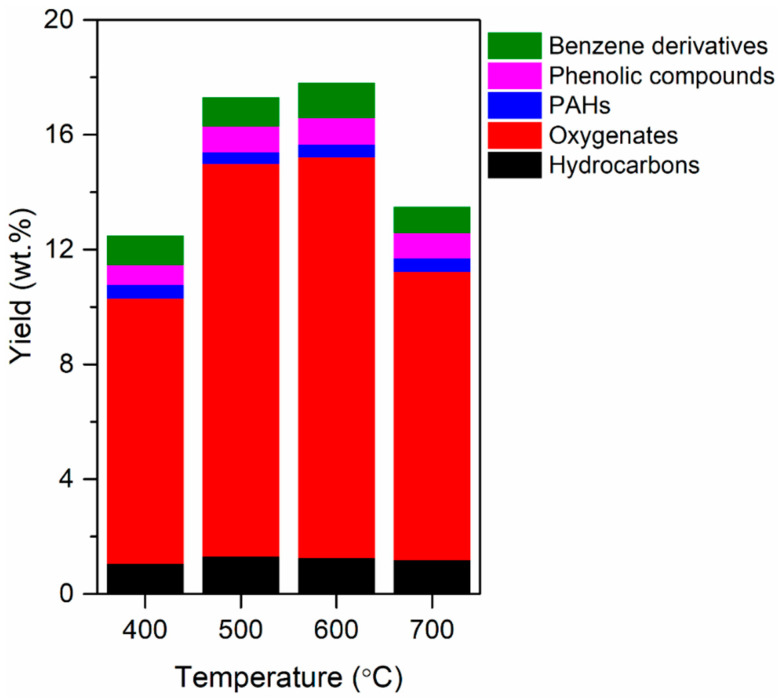
Yield of condensable compounds (on the basis of the weight of the feedstock) produced via the pyrolysis of TBW at different pyrolysis temperatures. Mean values of replicates (*n* = 3) are reported with standard deviations of approximately 7%.

Figure 3 shows the yields of the condensable species in the pyrolytic products (i.e., pyrolytic liquid). Figure S3 shows a representative GC—MS spectrum obtained via the condensable pyrolytic product analysis, representing the highest peak of caprolactam. The yield of total condensable species increased from 12.5 to 17.8 wt.% as the pyrolysis temperature increased from 400 to 600 °C. This was attributed to the decomposition of the polymeric structure of the biomass that occurs at temperatures above 400 °C [39,40]. A further increase in the temperature to 700 °C decreased the yield, likely due to the enhanced thermal cracking of condensable vapor (Figure 2).

As shown in Figure 3, the yield of phenolic compounds, benzene derivatives, and PAHs increased with an increase in temperature from 400 to 600 °C. Benzene derivatives and phenolic compounds are more easily transformed into PAH derivatives at higher temperatures, and the formation of PAHs favors high temperatures [41]. A further increase in the temperature from 600 to 700 °C decreased the yields of benzene derivatives, phenolic compounds, and PAHs, likely due to enhanced thermal cracking of those compounds.

Oxygenates that do not contain phenolic, benzene, and PAH functionalities were the most abundant species in the condensable pyrolytic product (Figure 3). The oxygenates were composed mainly of alcohols, aldehydes, ketones, acids, furans, and pyrans which are typically found in bio-oils [42,43]. This indicates that the oxygenated compounds originated from the biomass part of the waste tea bag (Figure S1 and Table 1).

**Figure 4:**
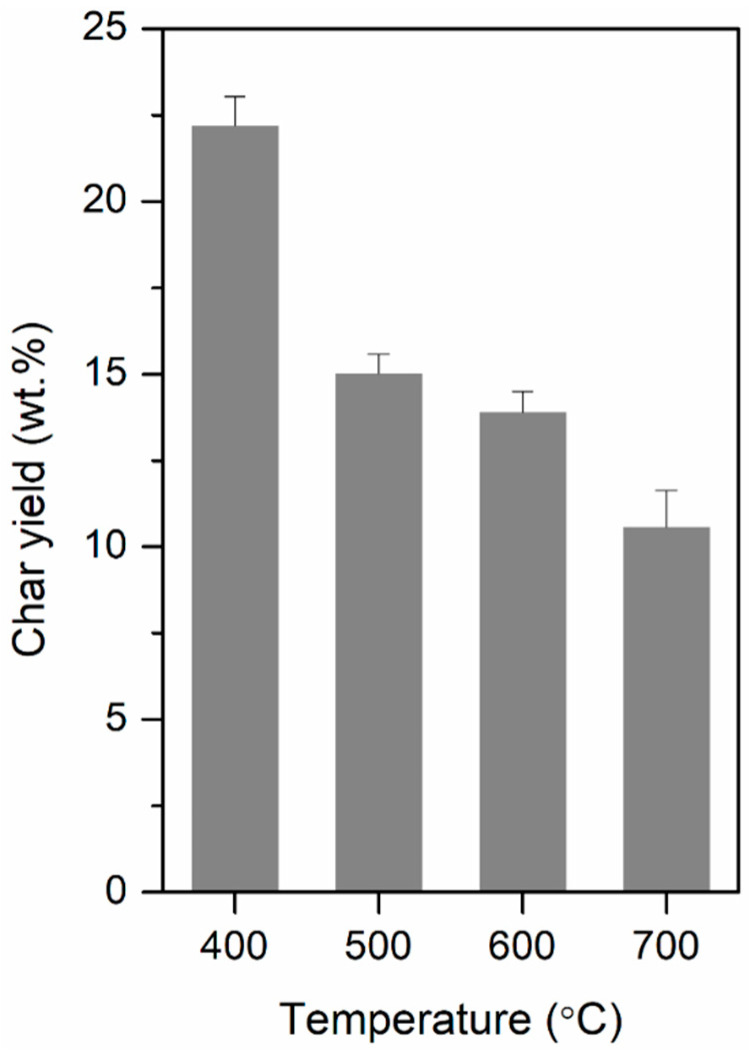
Yield of char (on the basis of the weight of the feedstock) produced via the pyrolysis of TBW at different temperatures. Mean values of replicates (*n* = 3) are reported with the standard deviations as error bars.

The yield of the nonvolatile carbon-rich solid remaining after the pyrolysis of the TBW (i.e., char) at different temperatures is shown in Figure 4. The yield of the char was the highest at 400 °C and decreased with an increase in the pyrolysis temperature. The decrease in the char yield was likely due to the enhanced cleavage of –OH and C–H groups at temperatures higher than 400 °C [44] which releases more volatile compounds from the feedstock [45], thereby decreasing the yield of the char.

**Figure 5:**
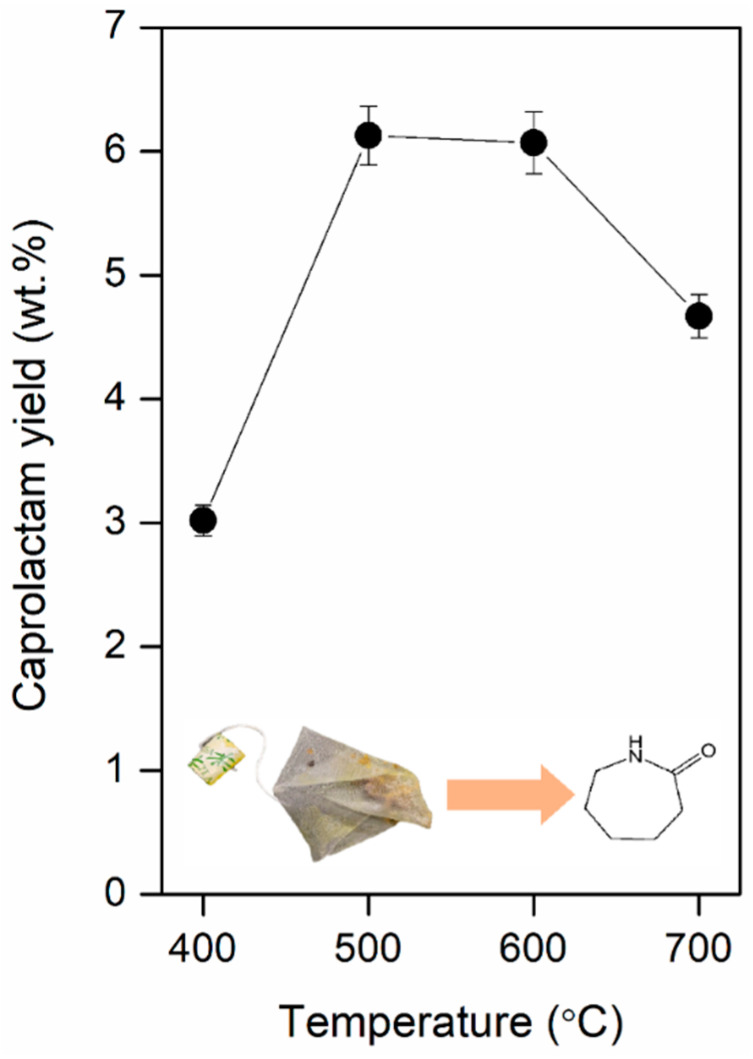
Yield of caprolactam (on the basis of weight of the feedstock) obtained by the pyrolysis of the TBW as a function of the pyrolysis temperature. Mean values of replicates (*n* = 3) are reported with the standard deviations as error bars.

Figure 5 shows the yield of caprolactam obtained from the pyrolysis of TBW conducted between 400 and 700 °C. The caprolactam yield increased from 3.1 to 6.2 wt.% as the temperature increased from 400 to 500 °C. However, a further increase in the temperature from 500 to 700 °C decreased the yield from 6.2 to 4.6 wt.%. This may be because the caprolactam is thermally decomposed at temperatures higher than 500 °C. Considering that the caprolactam originated from the non-biomass part of the TBW (Figure S1 and Table 1) via its depolymerization, and the caprolactam yield of 6.2 wt.% was on the basis of the weight of the whole TBW (i.e., both the non-biomass and biomass part), the caprolactam yield corresponds to 59.2 wt.% on the basis of the weight of the non-biomass part of the TBW.

The separation of caprolactam from the pyrolytic liquid would be important to commercialize the pyrolysis process for its recovery. However, in the liquid phase, there were many chemicals having similar boiling points to caprolactam. This made difficult apply the typical distillation method to separate caprolactam from the pyrolytic liquid. Therefore, future works on developing a method to separate caprolactam from the complex chemical mixture must be further conducted.

Pyrolysis is endothermic, so external heat is necessary to raise and maintain the temperature. Pyrolytic products such as combustible gases, pyrolytic liquid, and char resulting from the pyrolysis of the TBW can be used as fuels to supply external heat to the pyrolysis process. The HHV of the combustible gases produced at 500 °C (at which the highest caprolactam yield was achieved) was 7.7 MJ kg^−1^. The HHV of the pyrolytic liquid collected at 500 °C was 8.3 MJ kg^−1^. The HHV of the TBW-derived char made at 500 °C was calculated to be 23 MJ kg^−1^ using the elemental analysis results (Table S3). The HHV of the char is comparable to the HHV of coal (13.5 to 26.5 MJ kg^−1^) [46]. Thus, the char can be a solid fuel alternative to coal.

In many cases, TBW is put in food waste bins. Food waste collected in food waste bins is typically composted or digested; however, non-degradable part of TBW cannot be composted/digested. Therefore, in order to apply the pyrolysis process (that is suggested in this study) to treat TBW, TBW must be collected with being separated from food waste. As the pyrolysis process needs drying step; hence, the collected TBW should be dried preferably at the source.

## 4. Conclusions

To recover value-added chemicals (e.g., polymer monomers) from waste materials, we carried out the pyrolysis TBW in an N_2_ environment. Within the pyrolysis temperature range of 400 to 700 °C, higher temperatures led to the generation of more non-condensable gases and less char. The highest yield of caprolactam (6.2 wt.% per the whole TBW and 59.2 wt.% per the non-biomass part of the TBW) was achieved at 500 °C. The pyrolytic products of the TBW may be used as fuels to supply heat energy to the pyrolysis process. The HHVs of the pyrolytic combustible gases and the pyrolytic liquid were 7.7 and 8.3 MJ kg^−1^, respectively. The char derived from the TBW at 500 °C had a HHV of 23 MJ kg^−1^, indicating that it can be used as an alternative to coal. This work proves that pyrolysis can be used to recover value-added polymer monomers (e.g., caprolactam) from everyday waste such as TBW and to produce alternative fuels.

## Figures and Tables

**Table 1:** Results of proximate, ultimate, and composition analyses of the TBW.

Composition	Non-Biomass Part	Biomass Part
Proximate analysis (wt.%)		
Moisture	1.8	4.2
Volatile matter	96.5	76.6
Fixed matter	0.6	18.7
Ash	1.1	0.5
Total	100	100
Ultimate analysis (wt.%)		
C	68.0	49.7
N	4.7	0.3
H	10.7	7.5
O (by difference)	16.6	42.5
S	0	0
Total	100	100
Composition analysis (wt.%)		
Holocellulose	-	68.0
Lignin	-	28.2
Extractives	-	3.8
Total	-	100

## Supplementary Materials

The supplementary materials are available online at https://www.mdpi.com/2073-4360/12/11/2695/s1.
