# Supplementary material for: Pyrolysis for Nylon 6 Monomer Recovery from Teabag Waste
Source: Polymers (Basel). 2020 Nov 16;12(11):2695. doi: 10.3390/polym12112695 (PMC7697768; doi:10.3390/polym12112695)
Supplement: Supplementary file 1 [file polymers-12-02695-s001.pdf]

Supporting Information

Pyrolysis for Nylon 6 Monomer Recovery from Teabag Waste

Soosan Kim [1], Nahyeon Lee [2], Jechan Lee [1,2,3*]

[1] *Department of Environmental Engineering, Ajou University, 206 Worldcuo-ro, Suwon 16499, Republic of Korea*

[2] *Department of Environmental and Safety Engineering, Ajou University, 206 Worldcuo-ro, Suwon 16499, Republic of Korea*

[3] *Department of Energy Systems Research, Ajou University, 206 Worldcuo-ro, Suwon 16499, Republic of Korea*

[*]Corresponding author. jlee83@ajou.ac.kr (J. Lee)

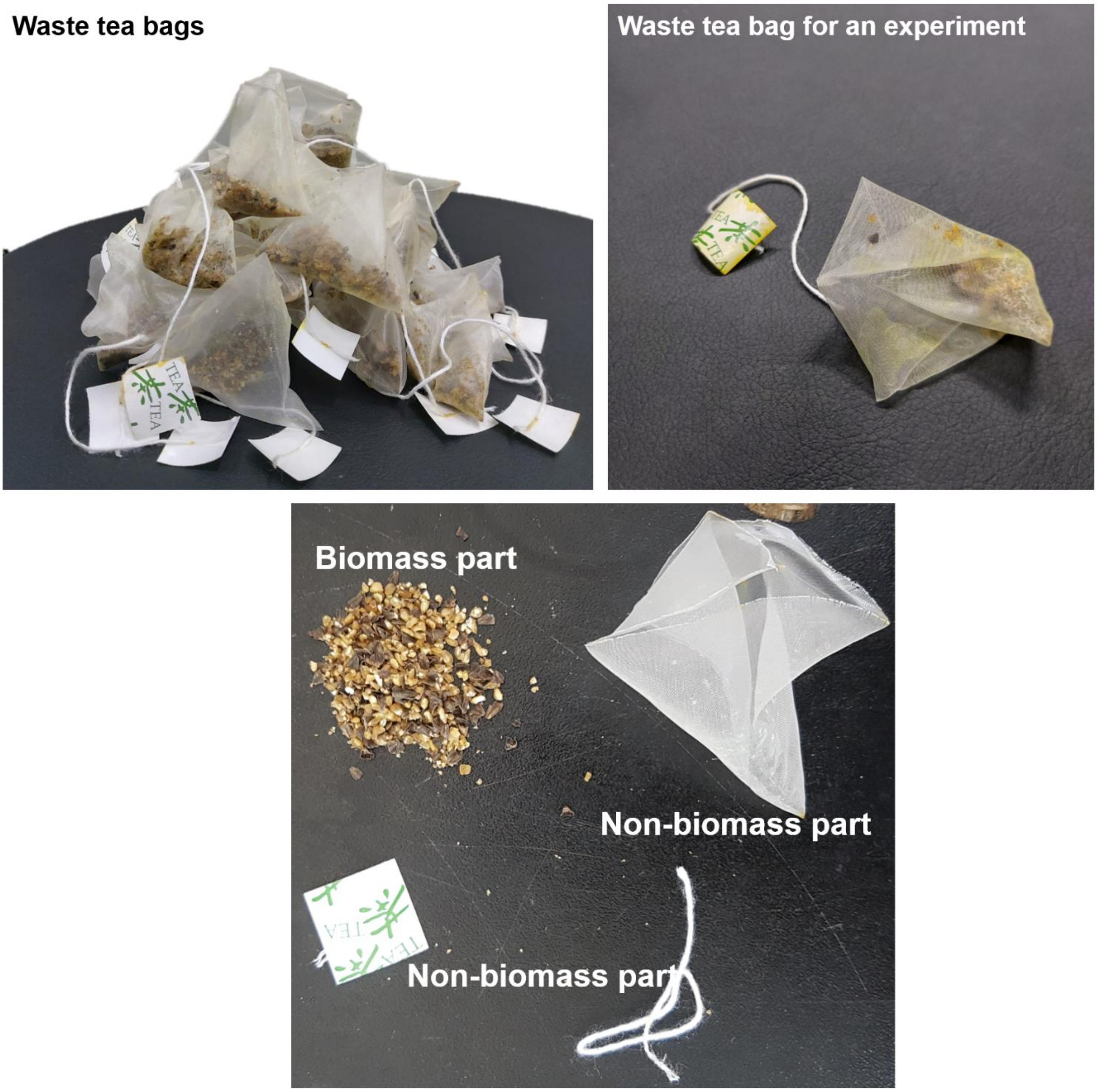

Figure S1. Waste tea bags used as the feedstock

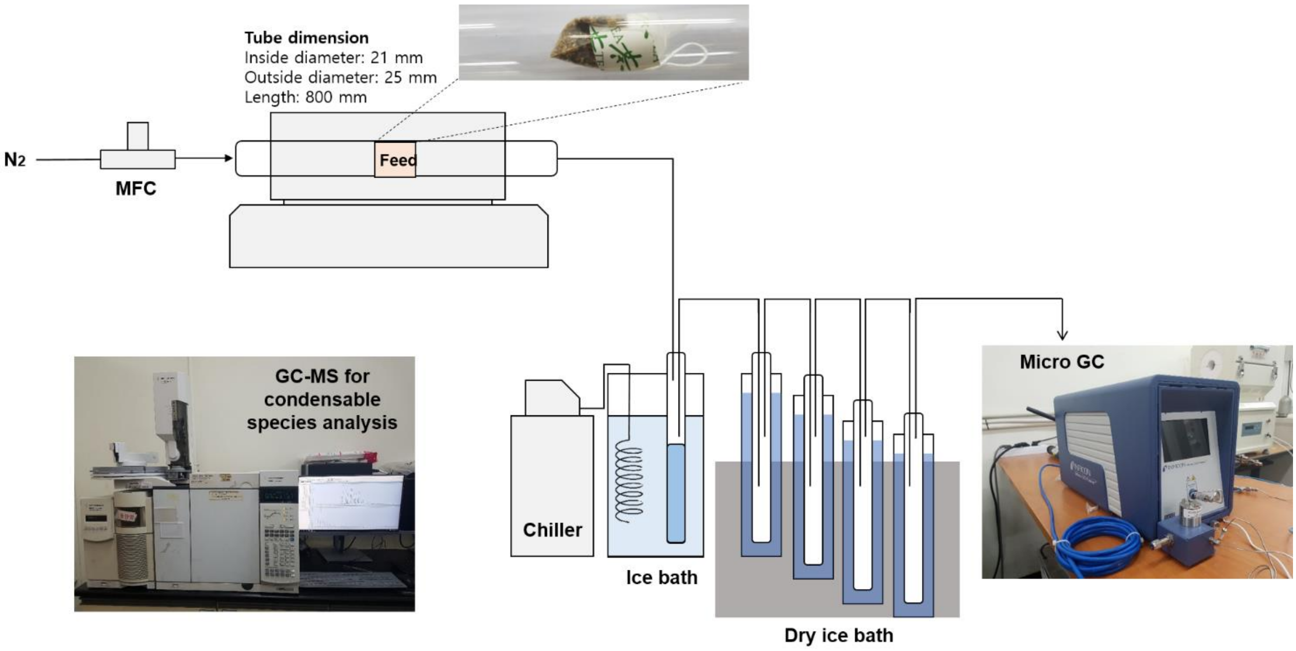

Figure S2. Schematic of the pyrolyzer used in this study

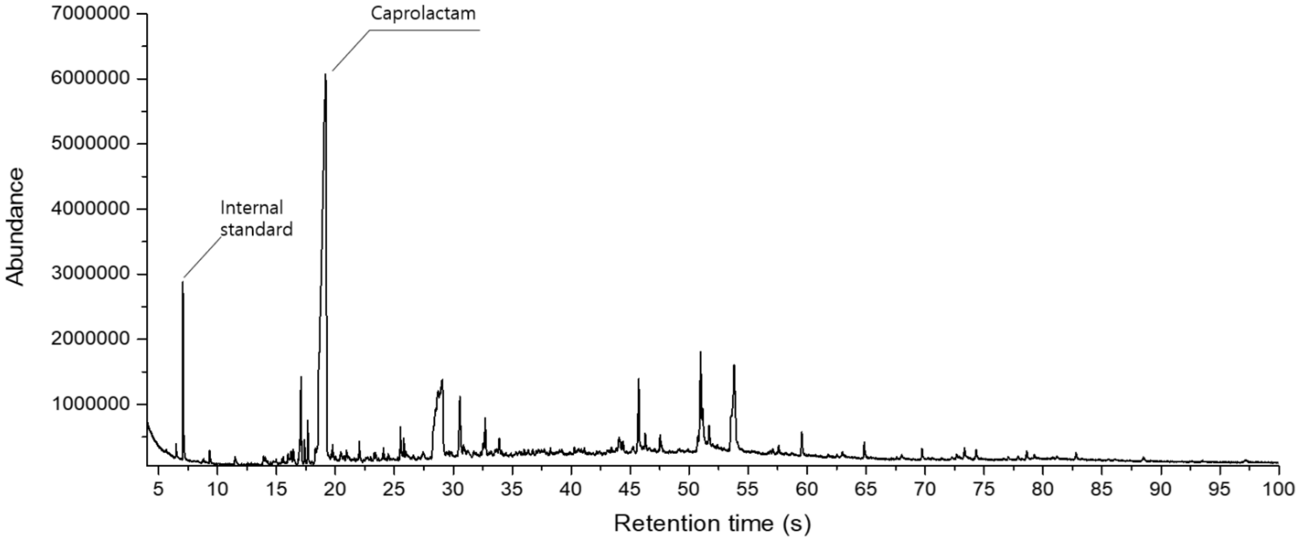

Figure S3. A representative GC–MS spectrum obtained via the condensable pyrolytic product analysis

Table S1. Specification, column information, and analytical conditions for the micro GC

Model		INFICON Fusion Gas Analyzer	
Conditions		Module A	Module B
Column		Rt-Molsieve 5A	Rt-Q-Bond
Sample Pump setting	Sample pump mode	Continuous	Continuous
	Sample pump time	20 s	20 s
Column setting	Carrier gas	Argon (≥99.999%)	Helium (≥99.999%)
	Column pressure	20 psi	17 psi
	Initial temperature	50 °C (40 s)	50 °C (30 s)
	Ramping time	50 s	60 s
	Final temperature	100 °C (40 s)	110 °C (40 s)
	Total analysis time	130 s	130 s
Injector setting	Inject time	30 ms	30 ms
	Injector temperature	90 °C	90 °C
TCD setting	TCD temperature	70 °C	70 °C
	Data rate	50 Hz	50 Hz

Table S2. Specification, column information, and analytical conditions for the GC–MS

Model	GC: Agilent 7890A; MS: Agilent 5975C	
Column	HP-5MS Ultra Inlet column (0.25 mm × 0.25 um × 30 m)	
Oven setting	Initial temperature	40 °C (1 min)
	Ramping	3 °C min^{-1}
	Final temperature	280 °C (19 min)
	Total analysis time	100 min
Column setting	Carrier gas	Helium (≥99.999%)
	Carrier gas flow	1.5 mL min^{-1}
	Column flow	1 mL min^{-1}
Injector setting	Injection mode	Splitless
	Injection volume	1 μL
	Injection temperature	275 °C
MS setting	Aux temperature	300 °C
	m/z range	50~500 amu

Table S3. Elemental composition and ash content of the char made at 500 °C

Elemental composition (wt.%)	N	3.65
	C	65.67
	H	2.27
	S	N.D.
	O (by difference)	23.09
Ash content (wt.%)		5.32
